# *Lactobacillus rhamnosus* Encapsulated in Alginate/Chitosan Microgels Manipulates the Gut Microbiome to Ameliorate Salt-Induced Hepatorenal Injury

**DOI:** 10.3389/fnut.2022.872808

**Published:** 2022-04-14

**Authors:** Zheng Zhang, Jiajian Liu, Mengjie Li, Binbin Yang, Wei Liu, Zhuangzhuang Chu, Bo Cui, Xiao Chen

**Affiliations:** ^1^State Key Laboratory of Biobased Material and Green Papermaking, School of Food Science and Engineering, Qilu University of Technology, Shandong Academy of Sciences, Jinan, China; ^2^College of Health Sciences, Shandong University of Traditional Chinese Medicine, Jinan, China; ^3^Yucheng People's Hospital, Dezhou, China; ^4^College of Natural Resources and Environment, South China Agricultural University, Guangzhou, China

**Keywords:** microgels, high-salt diet, gut microbiome, probiotic, hepatorenal injury

## Abstract

As the essential regulator of intestinal bacterial diversity, probiotics are a potential treatment for chronic high-salt diet (HSD)–induced metabolic dysfunction. Probiotic cells entrapped in microgels have been confirmed as being more effective than free cells in protecting bacteria against unfavorable conditions, that is, enhancing their stress resistance. This study explored the physiological mechanism by which probiotic microgels relieve HSD–induced hepatorenal injury. Herein, *Lactobacillus rhamnosus* was encapsulated in alginate-chitosan microgels which the percentage of alginate/chitosan was applied 1.5:0.5 (*w*/*w*) in this system, and the encapsulation significantly improved the probiotic viability in simulated gastrointestinal conditions. Mice were fed an HSD with *L. rhamnosus* (SDL) or *L. rhamnosus* microgels (SDEL). After 8 weeks of administration, dietary sodium was confirmed as inducing the hepatic and renal damages in mice, based on indicators, including serum biomarker levels, histopathological features of tissues, and pro-inflammatory cytokine contents in blood levels. However, the serum levels of urea nitrogen, creatinine, uric acid, glutamic-pyruvic transaminase, glutamic-oxalacetic transaminase, and alkaline phosphatase in the SDL and SDEL-fed mice were significantly lowered compared to the HSD-fed mice, especially in the SDEL group. HSD increased the abundances of *Anaeroplasma, Enterorhabdus, Parvibacter*, and *Bacteroides*, while the microgels increased the abundances of *Lactobacillus, Bifidobacterium, Mucispirillum*, and *Faecalibaculum*. Significant variations of fecal metabolome were validated for SDEL-treated mice, containing those linked to entero-hepatic circulation (e.g., cholic acid), carbohydrate metabolism (i.e., _L_-lactic acid), and increased antioxidants including citric acid. Furthermore, the probiotic microgels ameliorated intestinal damage by improving barrier and absorption functions. These results augmented existing knowledge on probiotic application for salt toxicity.

## Introduction

Probiotics are defined as living microorganisms that, when consumed in sufficient amounts, can exert beneficial effects on the host (1). For instance, probiotics can alleviate aging-related leaky gut (2), ameliorate autistic-like behaviors (3), reduce complications associated with colorectal cancer surgery (4), promote gastrointestinal symptoms in patients with celiac disease (5), and relieve constipation (6). Notably, healthy benefits can be gained from the oral intake of adequate amounts of living cells in quantities sufficient for survival through the upper gastrointestinal tract to the small intestine to colonize at the mucosal membrane of the colon. However, the viability of probiotics can be reduced during food preservation or handing through oxygen enrichment and/or thermal treatments (7). Encapsulation technology is one of the most effective methods available to improve the viability and stability of probiotics in fermented foods and the human gastrointestinal tract. Several materials have been investigated to encapsulate probiotics, such as polysaccharides (predominantly sodium alginate, chitosan, and starch), proteins, and lipids, which are employed to protect probiotics against the freezing and acid associated with the harsh environments present in commercial manufacturing, preservation, and oral delivery (8). Alginate and chitosan are naturally occurring biopolymers that are finding widespread applications in food and pharmaceutical industry. Alginate is used extensively in food industry as a thickener, emulsifier and as a stabilizer. Chitosan is a potentially useful pharmaceutical material owing to its good biocompatibility and low toxicity (9). Alginate and chitosan, by virtue of their physicochemical properties and mild gelation conditions, have gained the acceptance of researchers as matrices for the purpose of probiotics peroral delivery (10).

Chronic kidney disease (CKD) and renal dysfunction impose a tremendous burden on the healthcare system worldwide. It is widely considered that renal injury is caused by a suboptimal diet in humans, including high-salt diets (HSDs), high-fat diets, and/or excess foods (11–13). The human gastrointestinal tract provides habitat for more than 100 trillion commensal bacteria. Metagenomic analyses have revealed that CKD is highly correlated with intestinal dysbacteriosis, which may be linked to metabolites from the gut microbiota (14, 15). Renal excretory capability is an indispensable role of the host-microbial symbiosis. It enables intestinal absorption of the beneficial microbial metabolites, whereas the kidney removes useless and potentially harmful metabolites (16). This method of interorgan communication, closely processed through small molecule messengers, is regarded as remote sensing and signaling (17). Accumulating evidence from patients with CKD displays strong support for the kidney's role in host-microbial symbiosis (18, 19). Some of microbiota-derived metabolites, including indoxyl sulfate, deoxycholic acid (DCA), and trimethylamine oxide, were confirmed to accumulate in the blood parallel to the cause of the renal dysfunction development and proven to be related to clinical outcomes in patients with CKD (20–22). This paradigm has been coined the gut-kidney axis. Although probiotics have displayed beneficial effects on renal injury through modulating the intestinal microbiota modulation (23, 24), to date, the mechanism and impact of probiotics microgels on salt-induced renal impairment have not yet been elucidated.

Previously, research studies have validated that HSD could cause liver injury and affect the intestinal microbiota in mice, particularly reducing the abundance of *Lactobacillus* genus in the gut (25, 26). Additionally, oral treatment with *Lactobacillus* improved salt-induced experimental autoimmune encephalomyelitis and hypertension in mice (27). Considering these previous findings, we hypothesized that *L. rhamnosus* in microgels could effectively ameliorate salt-induced hepatorenal injury. Accordingly, *L. rhamnosus* was encapsulated in alginate/chitosan microgels, the physicochemical properties were analyzed, and they were used to treat the HSD mice in this study. After 8 weeks of treatment, the composition of the intestinal microbiota, fecal metabolites, serum biochemical indexes, hepatic and renal morphology, and intestinal function of mice was determined. The current research identifies mechanisms for the beneficial outcomes of *L. rhamnosus* in microgels on hepatorenal injury, and facilitating the development of probiotic supplements for treating CKD.

## Materials and Methods

### Bacterial Strain and Culture Conditions

*Lactobacillus rhamnosus* ATCC 7469 used in this study was obtained from the Guangdong Microbial Culture Collection Center (Guangzhou, China). *L. rhamnosus* stock solution was preserved in de Man, Rogosa and Sharp (MRS) broth supplemented with 25% glycerol (*v*/*v*) at −80°C and incubated aerobically in the fresh MRS broth medium for 12 h at 37°C. Cultures were centrifuged at 8,000 rpm for 10 min at 4°C, washed twice with normal saline solution (0.9% NaCl solution) to obtain cells, resuspended as bacterial cell pellets at 10^10^ colony-forming units (CFU)/mL and set aside for later use. In this study, the total viable bacteria were enumerated in triplicate using the drop-plate method after 2 days grown at 37°C on an MRS agar plate.

### Microencapsulation of *L. rhamnosus*

Bacterium encapsulation was performed using the method presented by Gao et al. (28) with some modifications. Briefly, sodium alginate solution of 1.5% (w/v) was prepared by being dissolved in normal saline solution and then filtered with a sterile membrane filter (0.22 μm pore size; Millipore, Bedford, MA, USA). Then 1 mL of resuspended *L. rhamnosus* solution, containing 10^10^ CFU, was centrifuged at 8,000 rpm for 5 min and suspended in sodium alginate solution. Approximately 1 mL suspension was extruded into the 1.1% (*w/v*) CaCl_2_ solution by an 0.5 mm-external diameter needle to shape the calcium alginate beads. The beads were hardened for 30 min and then transferred to 0.5% (*w/v*) chitosan solution at a beads/solution ratio of 1:10 (*v/v*) for 20 min to form an alginate-chitosan microcapsule membrane. The beads were then washed with normal saline solution to remove excess chitosan. Charges on the membranes were counteracted by adding a 0.15% alginate solution, coating the bead with another membrane for 20 min. Finally, the microcapsules were collected, washed, and transferred into 0.055 M sodium citrate for 5 min to harvest the microcapsules.

### Morphology of *L. rhamnosus* Microgels

Frozen microgels were freeze-dried at −40°C and 1 MPa in a vacuum dryer (LGJ-10, Songyuan Huaxing Technology Develop Co., Ltd., Beijing, China) for 48 h. Lyophilized *L. rhamnosus* microgels were mounted on aluminum specimen stubs, sputter-coated with a thin layer of gold, and investigated using scanning electron microscopy (SEM; Carl Zeiss EVO MA15-Smart SEM, Zeiss, Germany) under different magnifications.

### *In vitro* Simulated Digestion of *L. rhamnosus* Microgels

Simulated intestinal fluid (SIF) and simulated gastric fluid (SGF), which simulate digestive fluids in the gastrointestinal tract, were established as directed by the U.S.P. (https://www.usp.org). Briefly, 1 L SGF (pH 1.2) was prepared to contain 2 g NaCl, 3.2 g pepsin, 7 mL HCl, and deionized H_2_O. The stock intestinal solution was prepared including 6.8 g KH_2_PO_4_, 77 mL NaOH (0.2 M), and 0.5 L deionized H_2_O. Subsequently, 10 g pancreatin were mixed into the stock intestinal solution, with an adjusted pH of 6.8, and diluted with deionized H_2_O to 1 L to obtain SIF. Each 1 mL *L. rhamnosus* solution or the *L. rhamnosus* microgels (10^10^ CFU) was mixed separately with 9 mL of SGF, SIF, or 2% bile-salt solution, and digested in an incubator shaker at 37°C. The probiotic viability was determined at 0–60 min, using 10 min intervals. The probiotic amount of the microcapsules is quantified in log 10^10^ CFU/mL by sonicating 100 μL of the wet capsules in 1 mL of peptone water for 10 s using a Vibra-cell probe sonicator (VC 505, Sonics, USA). Then the viable bacteria were assessed using the drop-plate method after 2 days grown at 37°C on an MRS agar plate (29).

### Viability of Probiotics During Long-Time Storage

One-milliliter sample of free or encapsulated cells was mixed with 9 mL of sodium citrate after storing at 4°C for 8 weeks. After vortex mixing, samples were placed on a shaker at 37°C until microgels totally disintegrated. Viable bacteria were calculated by the plate count method on MRS agar in triplicate.

### Animal Intervention

Thirty-two male C57BL/6J mice (8-week-old; specific pathogen-free) were purchased from Pengyue Laboratory Animal Technology Co., Ltd. (Jinan, China) with production license number SCXK (Lu) 2019-0003. All mice were kept singly in clean conditions with a 12 h light/dark cycle and ample ventilation, at an ambient temperature of 24°C, and relative humidity of 50–70%. This study, approved by the Animals Ethics Committee of the Experimental Animal Center of Shandong University of Traditional Chinese Medicine (No. SYXKLU20170022, Jinan, China), complied with the EU Directive 2010/63/EU for the care and use of laboratory animals.

**Figure 1:**
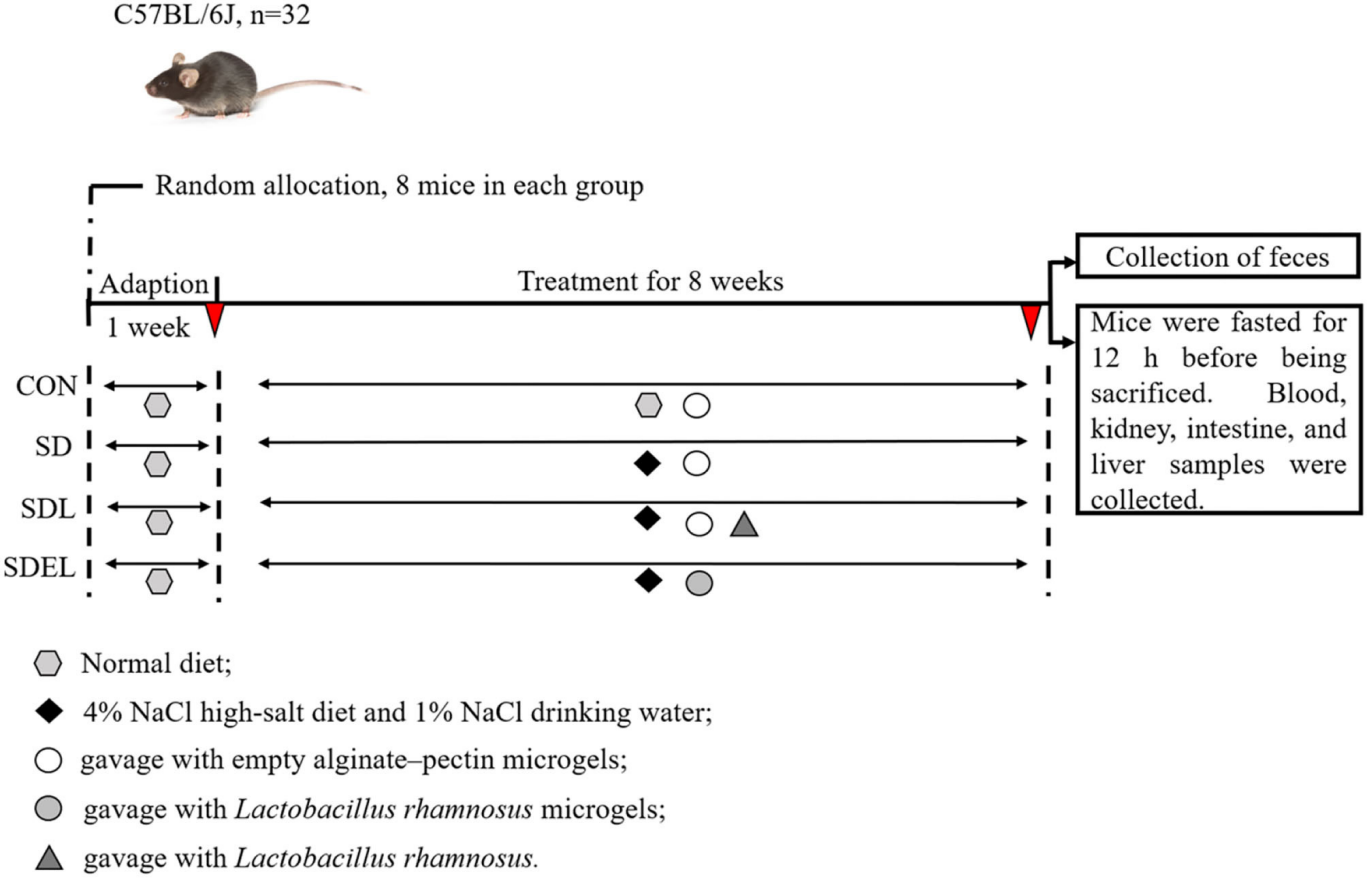
Animal and experimental design.

Mice were given a basic rodent-chow pellet diet and water *ad libitum* during the first week to enable acclimatization to the environment, followed by an 8-week administered diet (Figure 1). The animals were stochastically divided into four groups, each containing eight mice: CON group fed with a standard diet; SD group fed with 4% NaCl diet; SDL group fed with 4% NaCl diet and orally treated with 200 μL fresh *L. rhamnosus* suspensions (2 × 10^9^ CFU); and SDEL group fed with 4% NaCl diet and orally treated with 200 μL fresh *L. rhamnosus* microgels (2 × 10^9^ CFU) suspensions. Meanwhile, the mice in the CON, SD, and SDL groups were orally treated with the same volumes (200 μL) of the empty alginate-chitosan microgels suspensions. Furthermore, potable water for the SD, SDL, and SDEL mice was supplemented with 1% NaCl. All diets were sterilized by the 25 kGy γ-irradiation and prepared following the American Institute of Nutrition (AIN)-93G purified diet standard to satisfy the nutritional requirements for laboratory rodents. Fodder and potable water for the experimental animals were changed daily, and extra fodder was stored at −20°C. The animals were weighed weekly over the 8 weeks of diet intervention experiments.

### Sample Collection

The sampling methods were carried out essentially as described previously (30). At the end of the experimental period, the mice were not fed in the evening, and the next day, fresh feces and serum were collected, and the mice were sacrificed using cervical dislocation. After rinsing three times with sterile normal saline, renal, hepatic, colonic and small intestinal tissues were fixed using fresh 4% paraformaldehyde solution. After sectioning, these tissues were assessed with hematoxylin and eosin (H&E) staining to observe the pathological changes under a microscope. In addition, the kidney and liver tissues were investigated by Masson staining to evaluate the histology of organ fibrosis, and the pathological scores of kidney and liver tissues were calculated per the method of Fang et al. (31).

### Gut Microbiota Profiling

The purity of DNA was isolated from fecal samples by following the QIAamp DNA stool mini kit protocol (Qiagen, GmbH, Hilden, Germany). Processing of separated DNAs, Illumina MiSeq amplicon sequencing, and procedures for library generation were performed according to the general method described previously (30). The V4–V5 regions of the bacterial 16S rRNA gene sequences were amplified using the system of polymerase chain reaction (PCR) containing the specific primers (515F: 5′-GTGCCAGCMGCCGCGG-3′ and 907R: 5′-CCGTCAATTCMTTTGAGTTT-3′) and a HiFi Hot Start Ready Mix (KAPA Biosystems, Woburn, MA, USA). Amplicons from the second PCR round were purified, pooled, and paired-end (PE) sequenced.

The raw Illumina paired-end reads in FASTQ format were quality trimmed using Trimmomatic software (32) to detect and cut off ambiguous bases and then matched using FLASH software (33) following the parameters as previously reported (25). The Quantitative Insights into Microbial Ecology (QIIME, version 1.8.0) pipeline was employed to process sequence data with 75% of the bases showing quality scores above 20 (base-calling accuracy of 99%) (34). All effective tags were clustered into operational taxonomic units (OTUs) at a 97% stringency threshold using the workflow provided by the QIIME wrappers (35). All representative reads were identified and classified into taxonomic levels using a nucleotide Basic Local Alignment Search Tool comparison against the Greengenes (16S rRNA) database and the classifier tool of the Ribosomal Database Project II Classifier with a confidence score threshold of 70% (36).

### Fecal Metabolomic Profiling

In brief, fecal pellets were spiked with 20 μL of an L-2-chlorophenylalanine solution (1 mg/mL in distilled water) as an internal standard and the metabolites were extracted with chloroform and methanol. After homogenization and bath sonication, the supernatant liquid was transferred into a fresh glass vial. The microbial-host co-metabolites were analyzed using an Agilent 7890A GC system (Agilent Technologies, Santa Clara, CA, USA) coupled to a Pegasus HT time-of-flight mass spectrometry (Leco, Saint Joseph, MI, USA) following the parameters as previously reported (26).

### Analysis of Serum Biochemical Indexes and Intestinal Function

Biochemical indicators in serum, including blood urea nitrogen (BUN), creatinine (CRE), uric acid (UA), glucose (GLC), glutamic-pyruvic transaminase (ALT), glutamic-oxalacetic transaminase (AST), alkaline phosphatase (ALP), and triglycerides (TG), were evaluated on an automatic biochemical analyzer (Au680, Beckman Coulter, Inc., Brea, CA, USA). The serum interleukin (IL)-17a and IL-22 levels were measured using enzyme-linked immunosorbent assay (ELISA) kits (Gersion Bio-Technology Co., Ltd., Beijing, China) according to the manufacturer's protocol.

To assess the intestinal barrier function, the serum levels of _D_-lactate (_D_-LA) and diamine oxidase (DAO) were analyzed using the respective ELISA kits (Gersion Bio-Technology Co., Ltd.) according to the manufacturer's protocol. The morphological development of the small intestine, including crypt depth and villus height, were measured using Image Pro-Plus version 6.0 software (Media Cybernetics, Inc., Rockville, MD, USA) to analyze the absorptive function. The pH value of the colonic contents was determined as previously described (26).

### Data Analysis

All results are represented as the mean ± standard deviation (SD) of replicate tests or as the median with interquartile ranges. As appropriate, the *t*-tests were performed to analyze the independent measurements using SPSS version 22.0 software (SPSS Inc., Chicago, IL, USA). One-way analysis of variance was used to analyze the differences between groups; multiple groups were compared with the Turkey *post-hoc* test. Significance was established at *P* < 0.05. All data analyses were run in R-statistical software (version 2.13.0; Free Software Foundation, Boston, MA, USA). Multivariate analyses [principal co-ordinates analysis (PCoA)] was performed using the Simca-P 14.1 software package (Umetrics, Umea, Sweden).

## Results

### Characterization of *L. rhamnosus* Microgels *in vitro*

**Figure 2:**
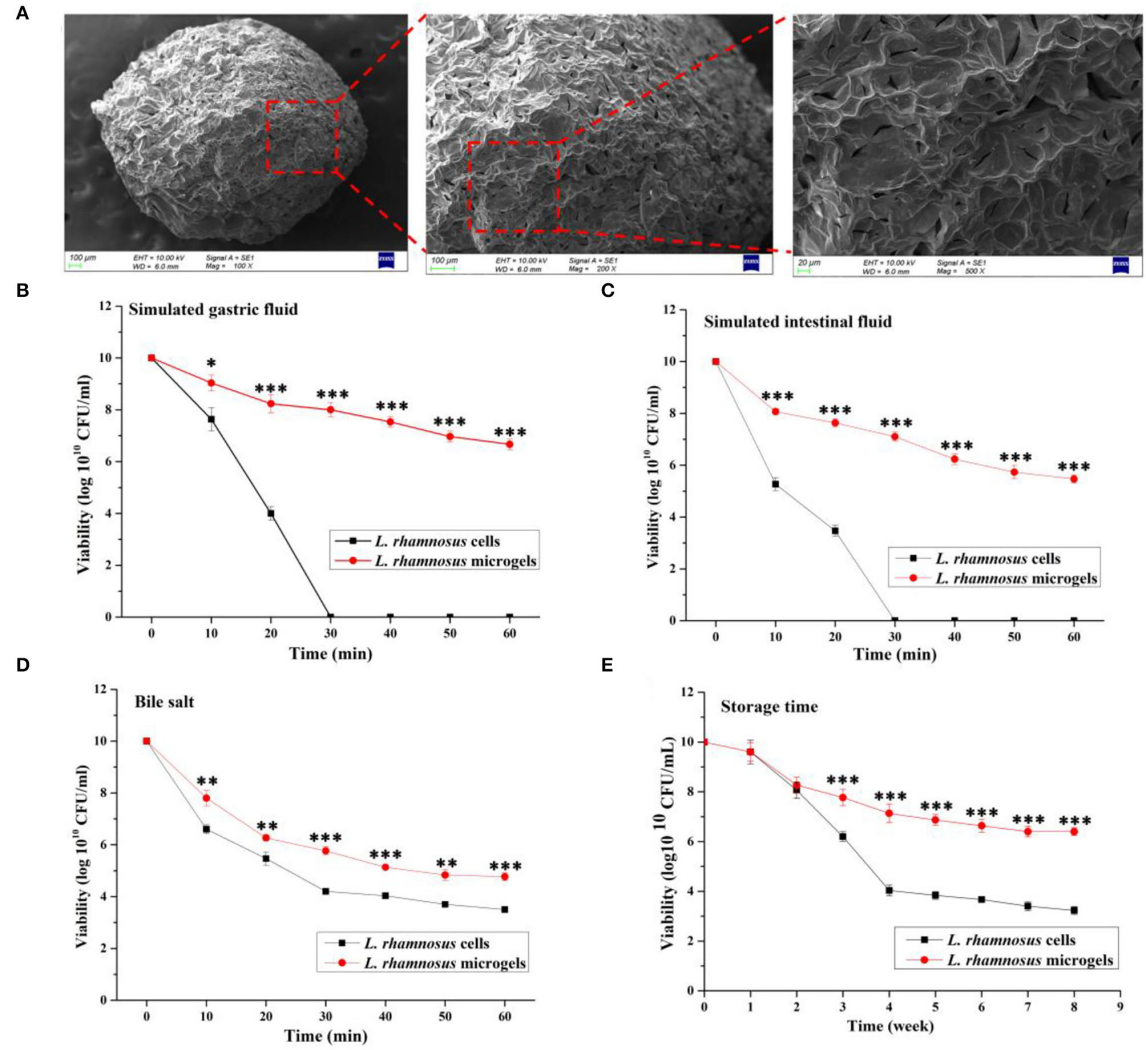
Morphology and characteristics of *L. rhamnosus* microgels. **(A)** The freeze-dried *L. rhamnosus* microgels were characterized using scanning electron microscope. The viability of *L. rhamnosus* microgels in a simulated gastrointestinal environment was separately assessed in **(B)** simulated gastric fluid, **(C)** simulated intestinal fluid, and **(D)** bile salt. **(E)** Anti-inactivation of *L. rhamnosus* microgels were analyzed in 8 weeks storage. Values are presented as the mean ± SD; *n* = 3. ^*^P < 0.05, ^**^P < 0.01, ^***^P < 0.001 vs. *L. rhamnosus* microgels.

In this section, SEM was employed to inspect the morphology of the *L. rhamnosus* microgels (Figure 2A). The SEM results show that the freeze-dried microgels were regularly olive-shaped, and the surfaces of the microgels appeared shrunken and wrinkled. Small cracks and high porosity could be found at the rough surface of microgels under higher magnification, consistent with a previous finding (37). The viability of bacterial cells is decreased by acidic and alkaline digestive fluids, enzymes, and bile salts in the host's gastrointestinal tract. We evaluated the effect of encapsulation of cell viability under simulated gastrointestinal environments to mimic these harsh conditions and calculate the survival rates of free or encapsulated probiotic cells. The results indicated that in both free and encapsulated cells, the viability of bacterial cells was reduced in simulated *in vivo* conditions. The viability of free probiotic cells was lost after 30 min exposure in both SGF and SIF (Figures 2B,C). The effect of encapsulation on *L. rhamnosus* viability in long-term storage was evaluated by storing freeze-dried samples at 4°C for 8 weeks to simulate commercial storage. The viability of free or encapsulated probiotic cells did not significantly differ at the first and second week in storage (*P* > 0.05). Subsequently, the viability of free probiotic cells was significantly decreased than that of encapsulated probiotic cells at the third week to 8 week (*P* < 0.001; Figure 2E).

Interestingly, the viability of the probiotics encapsulated in alginate/chitosan microgels was greatly improved, with higher resistance to gastric fluids. The viability of *L. rhamnosus* in microgels was 6.66 ± 0.21 and 5.47 ± 0.15 log10^10^ CFU/mL at 60 min exposure in SGF and SIF, respectively (Figures 2B,C). The viability of *L. rhamnosus* in microgels (4.77 ± 0.15 log10^10^ CFU/mL) exposed to 2% bile salt for 60 min was significantly increased over that of the free probiotic cells (3.50 ± 0.10 log10^10^ CFU/mL) (*P* < 0.05, Figure 2D). Therefore, the *L. rhamnosus* cells were successfully encapsulated in alginate/chitosan microgels, and the microgels improved the viability and resistance of bacterial cells in simulated gastrointestinal environments.

### Analysis of Hepatorenal Injury

**Figure 3:**
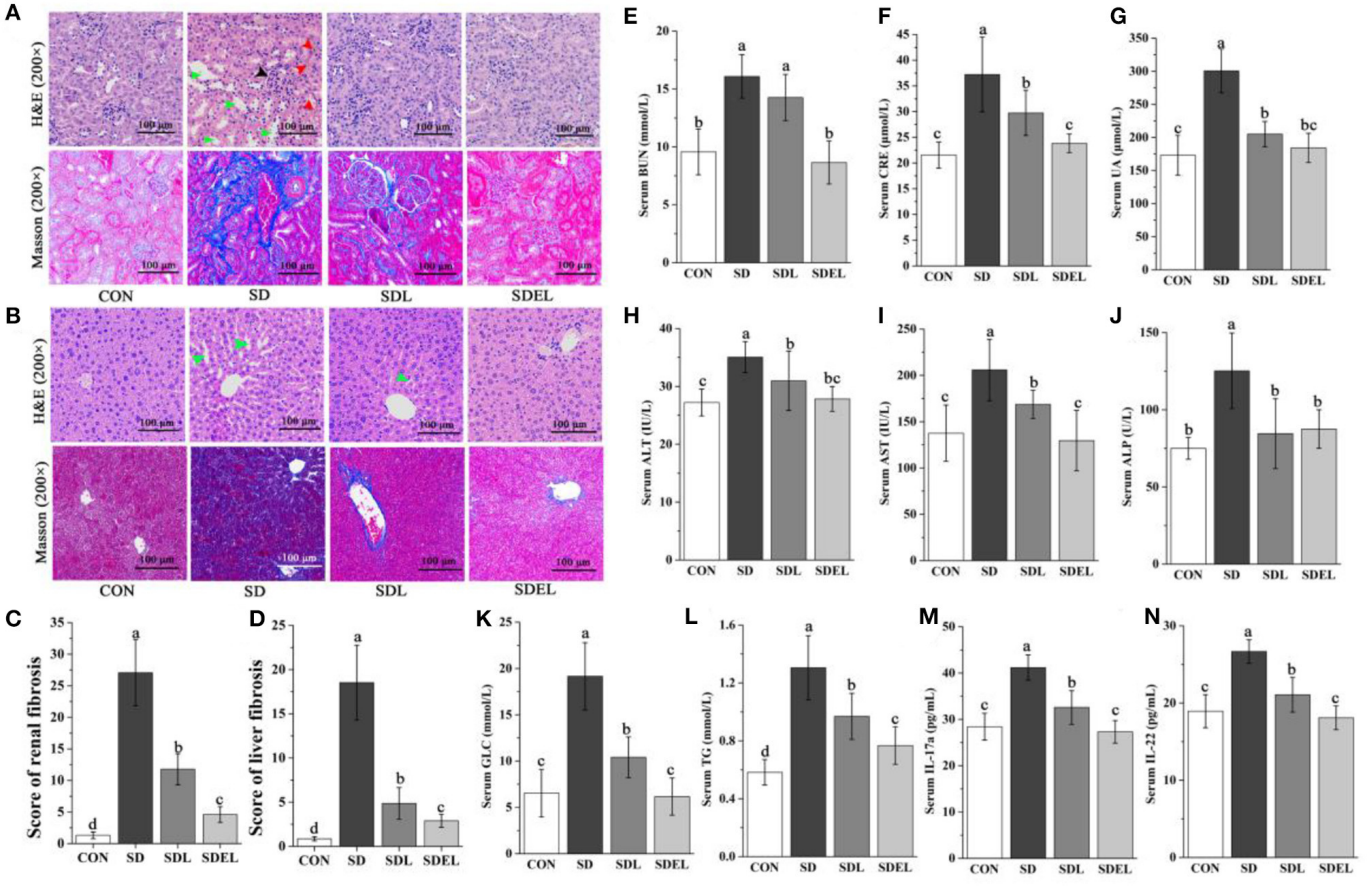
*L. rhamnosus* and *L. rhamnosus* microgels administration attenuated HSD-induced hepatorenal injury. **(A)** Representative kidney sections with H&E and Masson staining (200× magnification). The black arrow indicates vacuolations and swelling of glomerular cells, red arrows indicate interstitial angiectasis hyperemia, and green arrows indicate edema in renal interstitial spaces. **(B)** Representative liver sections with H&E and Masson staining (200× magnification). Green arrows indicate the loss of cellular boundaries around the central vein of hepatocytes. Compare **(C)** the renal fibrosis and **(D)** the liver fibrosis scores in various groups. Serum levels of **(E)** BUN, **(F)** CRE, **(G)** UA, **(H)** ALT, **(I)** AST, **(J)** ALP, **(K)** GLC, **(L)** TG, **(M)** IL-17a, and **(N)** IL-22 were determined in different groups. Data are expressed as mean ± SD; *n* = 8. Different letters were significantly different (*P* < 0.05).

After 8 weeks of supplementation, biomarkers of renal function (serum BUN, CRE, and UA contents) and liver injury (serum AST, ALT, and ALP activities) were measured. The results showed that the serum levels of BUN (Figure 3E), CRE (Figure 3F) and UA (Figure 3G) in the SD group were significantly increased compared with those in the CON group (*P* < 0.05), confirming that dietary salt-induced the renal dysfunction occurred in the SD-fed mice. Likewise, the serum ALT (Figure 3H), AST (Figure 3I), and ALP (Figure 3J) activities in the SD group were significantly increased compared with those in the CON group (*P* < 0.05), confirming that impaired liver function existed in the SD-fed mice. Additionally, the serum concentration of glycolipid (including GLC and TG) and inflammatory factors (e.g., IL-17a and IL-22) in the SD group were considerably increased compared with those of the CON group (*P* < 0.05, Figures 3K–N). The treatment of the SD-fed mice with *L. rhamnosus* or its microgels significantly reduced all of the serum biochemical parameters levels. Notably, the BUN, CRE, AST, GLC, TG, IL-17a, and IL-22 levels in the SDEL were significantly decreased compared with those in the SDL group (*P* < 0.05). Finally, there were no statistically significant differences in the mice's body weights among the four groups throughout the experimental period (*P* > 0.05; Supplementary Figure 1).

We used H&E staining to examine the histological changes of pathological tissue morphology and glomerular volume, as well as Masson staining to show the degree of fibrosis (Figures 3A,C). The severity of the tissue lesions varied among the four groups. The CON group showed structural integrality in renal tissue cells, normal appearance of the hepatic lobular structure and central vein, and no changes in glomerular morphology. In the SD group, the renal tissues appeared to have undergone some definitive pathological changes, including vacuolations and swelling of glomerular cells (black arrow), interstitial angiectasis hyperemia (red arrows), edema in renal interstitial spaces (green arrows), and severe renal tubule interstitial fibrosis. Treatment of SDL and SDEL mice could significantly curtail these adverse changes in the SD group. In particular, following SDEL intervention, swelling of the glomerular cells was alleviated. The nuclei were typical, and interstitial angiectasis hyperemia decreased. In cross-sections, the appearances of the kidney, glomerulus, and renal tubule structure were regular, and renal tubule interstitial fibrosis levels were restored significantly.

The H&E and Masson staining methods were also employed to observe the micromorphology of the liver tissues (Figures 3B,D). In the CON group, the liver showed a typical structure, with the nucleus located in the middle of the hepatic cells, no apoptosis, and hepatocytes arranged as cords and distributed uniformly. In the SD group, the hepatic cords were dissociated loosely and randomly, and there was neutrophilic granulocyte infiltration in hepatocytes, loss of cellular boundaries around the central vein of hepatocytes (green arrows), and prominent fibrosis in the liver tissue. As expected, the hepatic injury was effectively ameliorated to varying extents after 8 weeks of treatment with *L. rhamnosus* or *L. rhamnosus* microgels. In comparison with SDL-fed mice, the liver cells of the SDEL-fed mice were more evenly arranged with clear nuclei, no swollen cytoplasmic vesicular, and no fibrosis in liver tissues. Briefly, treatment with *L. rhamnosus* microgels significantly mitigated the hepatorenal injury of SD-fed mice.

### Analysis of Fecal Microbiota

**Figure 4:**
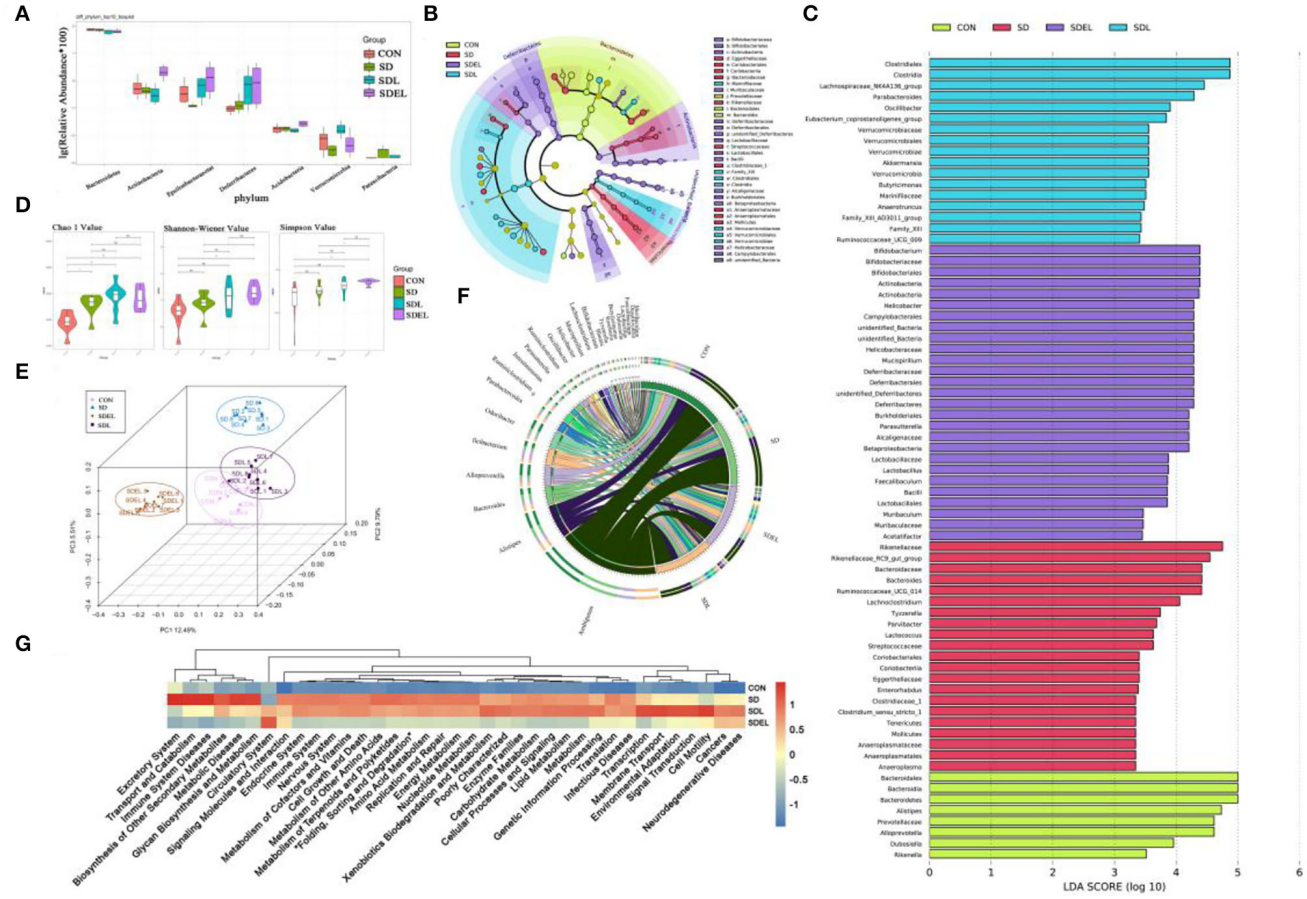
**(A)** Relative abundance of seven phyla in the CON, SD, SDL, and SDEL groups: Bacteroidetes, Actinobacteria, Epsilonbacteraeotat, Deferribacteres, Acidobacteria, Verrucomicrobia, and Patescibacteria. **(B)** Cladogram of linear discriminant analysis effect size (LEfSe) analysis identified the most differentially abundant taxa of gut microbiota in the different groups. **(C)** Histogram of LDA scores by comparing inter-group variance at the relative abundance level by LEfSe analysis. **(D)** Microbial richness estimates (Chao index) and diversity indices (Shannon–Wiener and Simpson) in the different group at the 8-week point. **(E)** PCoA shows separation of the fecal bacterial composition of the different groups. **(F)** The chord diagram reveals the four groups' top 25 abundant genera (abundance >0.1%). **(G)** Microbial community functions predicted by PICRUSt of the different groups.

After removing unqualified sequences, the CON, SD, SDL, and SDEL groups yielded more than 51,309, 69,761, 71,065, and 71,732 effective tags, respectively. Species were annotated with representative 16S rRNA gene sequence fragments. The effective sequences of all samples qualified OTUs were clustered based on ≥97% sequence identity. The box-and-whisker plot (Figure 4A) shows the relative abundances of seven major phyla analyzed to be significantly different (*P* < 0.05) among the CON, SD, SDL, and SDEL groups, as follows: Bacteroidetes, Actinobacteria, Epsilonbacteraeotat, Deferribacteres, Acidobacteria, Verrucomicrobia, and Patescibacteria. Linear discriminant analysis effect size (LEfSe) identified the differentially abundant bacterial genera that were differentially abundant in the four groups. At the family level, Eggerthellaceae, Bacteroidaceae, Rikenellaceae, Clostridiaceae_1, and Anaeroplasmataceae, as the biomarkers, were more abundant in the SD group. In contrast, Bifidobacteriaceae, Muribaculaceae, Deferribacteraceae, Lactobacillaceae, Alcaligenaceae, and Helicobacteraceae were more abundant in the SDEL group (Figure 4B). At the genus level, *Anaeroplasma, Enterorhabdus, Lactococcus, Parvibacter, Tyzzerella, Lachnoclostridium*, and *Bacteroides*, as the biomarker, were more abundant in the SD group, while *Lactobacillus, Bifidobacterium, Parasutterella, Helicobacter, Mucispirillum, Faecalibaculum, Muribaculum*, and *Acetatifactor* were more abundant in the SDEL group (Figure 4C). The α-diversity—which consists of richness estimates (i.e., Chao 1 index) and diversity values (including Shannon and Simpson indices)—of the microbial community were measured. The richness estimates of the microbial community in the SD, SDL, and SDEL groups was significantly higher than that of the CON group, while the diversity values of the microbial community in CON and SD groups was lower than that of the SDEL group (Figure 4D). PCoA indicated the bacterial diversity among the four groups; 27.79% of the total variance was attributable to the three principal components (PC1, PC2, and PC3), which were stable and reliable. The chord diagram reveals the top 25 abundant genera in the four groups (Figure 4F), showing that *Ambiguous, Alistipes*, and *Bacteroides* were the three predominant taxa in all the groups, accounting for 2.51–24.25% of the total OTUs. The differential abundance among the four groups showed that the *L. rhamnosus* microgels caused variation, for instance, in the levels of hepatorenal injury and the structure of the mouse gut microbiome. The discrepancy of functional profiles between different groups predicted by PICRUST (38). The results showed the overall differences in KEEG abundances among the microbiomes and revealed that biosynthesis of other secondary metabolites, metabolic diseases, immune system diseases, glycan biosynthesis and metabolism, transport and catabolism, excretory system, metabolism of other amino acids, metabolism of cofactors and vitamins, carbohydrate metabolism, and lipid metabolism in the SDEL group was significantly lower than that in the SD group (Figure 4G; *P* < 0.005).

### Metabolic Functionality

**Figure 5:**
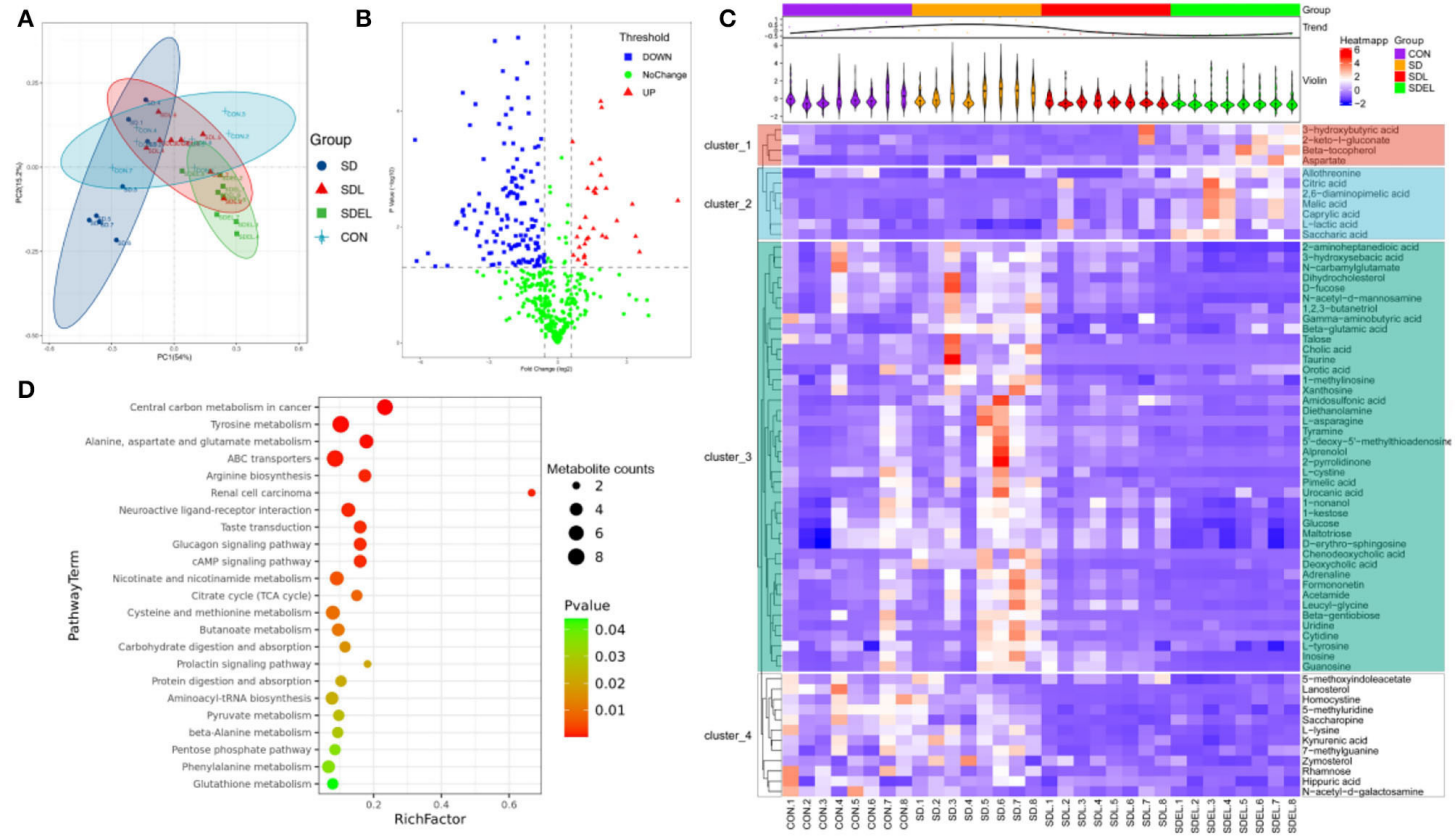
Fecal metabolic profiling of the CON, SD, SDL, and SDEL groups. **(A)** Scatterplot of PCoA scores in different groups. **(B)** Volcano plot showing the differential variables between the SD and SDEL groups. Each metabolite is represented by a dot, where the red dots indicate up-regulation, the blue dots indicate down-regulation, and the green dots indicate no statistical difference. **(C)** Heatmap of 70 metabolites at normalized levels differentially abundant (*P* < 0.05) among the three groups. The dendrogram shows the distances of the metabolites based on their relative abundances. The normalized abundance values are depicted visually from red to blue, representing the highest and lowest abundances. **(D)** Enriched KEGG pathways in the SD group compared with the SDEL group. The statistical significance values (*P* < 0.05) are depicted visually from red to green, representing the most and least differences, respectively. The dot sizes on the vertical axis represent the metabolite counts in the metabolic pathways.

Fecal metabolic profiling is a functional readout of gut microbiota that enables comprehension of the possible mechanisms underlying how the microbiota influences host health (39). A total of 592 peaks were identified, and 468 metabolites persisted after eliminating background noise using the interquartile range denoising method. Missing raw data values were generated using half of the minimum value. PCoA (Figure 5A) illustrates the data distribution and the separation in the metabolic compositions of the different groups. Specifically, 69.20% of the total variance resulted from PC1 PC2, and PC3, which were stable and reliable. The variables by which the content differed between the SD and SDEL groups are shown in the form of a volcano plot (Figure 5B), wherein each dot represents a metabolite: red dots represent upregulated metabolites, blue dots represent downregulated metabolites, and green dots represent metabolites with non-significant differences (*P* > 0.05). However, the volcano graph was complex since the various variables were included. Consequently, 70 differential metabolites between the SD and SDEL groups were identified based on the variable importance in the projection (VIP) value (>1.0) of the orthogonal partial least squares discriminant analysis and the Student's *t*-test *P*-value (< 0.05). The heatmap visualization (Figure 5C) was used to show differential metabolites, thus refining the analysis. A total of 58 metabolites, such as cholic acid, glucose, uridine, kynurenic acid, urocanic acid, hippuric acid, and _L_-tyrosine, exhibited significantly decreased abundance in the feces of SDEL-treated mice, whereas the abundance of 12 metabolites, such as _L_-lactic acid, 3-hydroxybutyric acid, saccharic acid, aspartate, citric acid, and β-tocopherol was significantly increased compared to levels in the feces of SD-fed mice. In particular, the abundance of _L_-lactic acid in the SDEL group increased the most, more 3.65 times than that of the SD group (Supplementary Table 1).

The significantly different metabolites occurring in the SD and SDEL groups were assigned based on metabolic pathways present in the Kyoto Encyclopedia of Genes and Genomes (KEGG) database. This analysis suggested that the differentially abundant metabolites in the SDEL group were all associated with central carbon metabolism in cancer, ABC transporters, arginine biosynthesis, tyrosine, alanine, aspartate and glutamate metabolism as well as renal cell carcinoma pathways (*P* < 0.01, frequency-distance relationship correction; Figure 4D).

### Integration of Among Metabolites, Bacteria, and Clinical Chemistry Data

**Figure 6:**
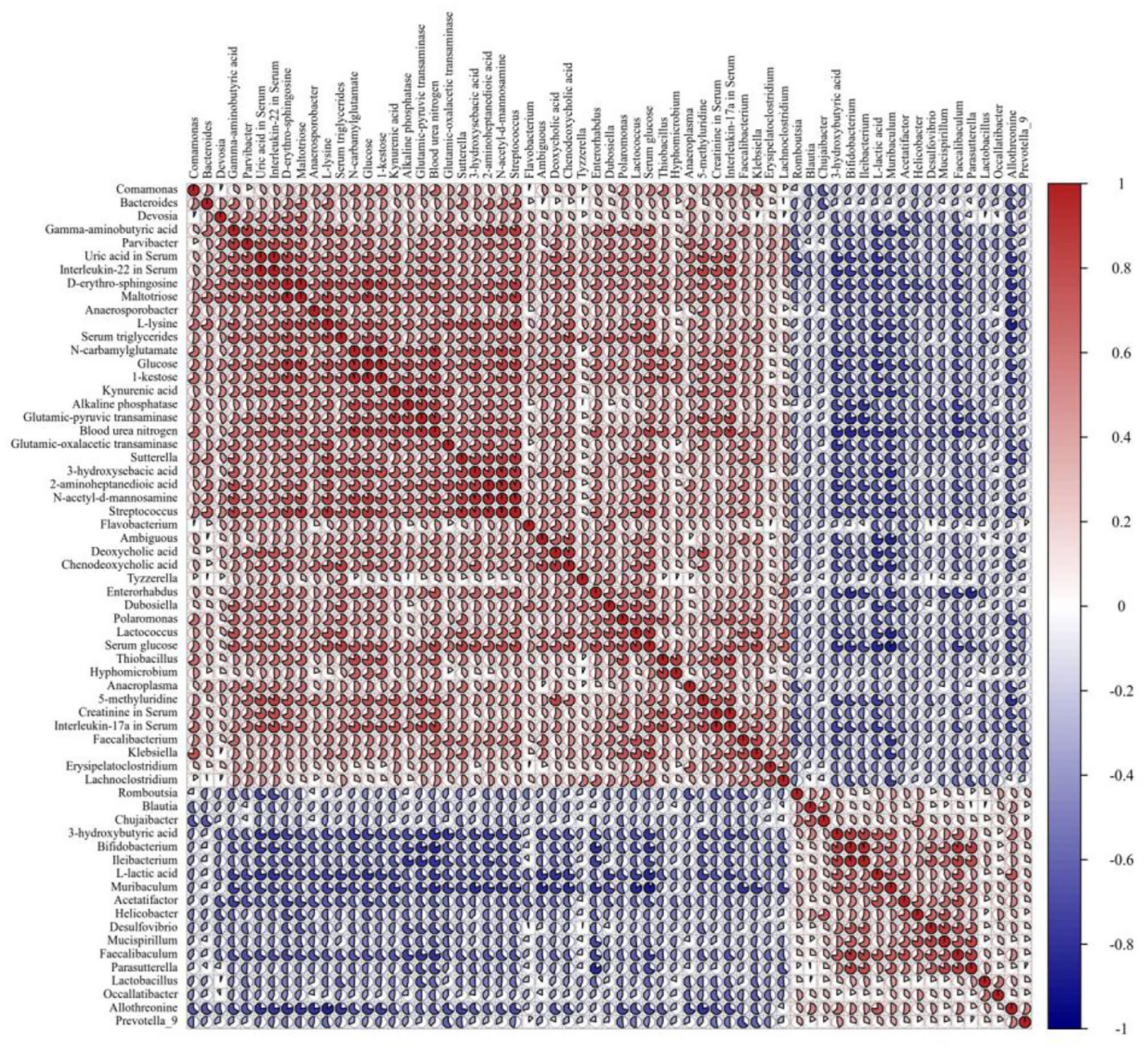
The Pearson correlation analysis among the 36 gut bacteria (*P* < 0.05), 17 fecal metabolites (*P* < 0.001), and 10 clinical chemistry data (*P* < 0.05) in the SD group compared with the SDEL group. *R*-values, the sector area, are depicted from blue to red, where red represents positive correlations and blue negative correlations.

Pearson's correlation analysis was employed to determine the correlations among gut microbiota differentially metabolites, and serum biochemical parameters. The resulting metabolic association heatmap (Figure 6) displays positive and negative correlations among the identified genera, the metabolite levels, and the clinical chemistry data. The dominant genera in SD group, including *Anaeroplasma, Enterorhabdus, Lactococcus, Parvibacter, Tyzzerella, Lachnoclostridium*, and *Bacteroides*, displayed strong positive correlations with _D_-erythro-sphingosine, glucose, kynurenic acid, maltotriose, N-acetyl-d-mannosamine, _L_-lysine, 5-methyluridine, 2-aminoheptanedioic acid, N-carbamylglutamate, deoxycholic acid, 3-hydroxysebacic acid, and all of the serum biochemical parameters. The dominant genera in the SDEL group, including *Lactobacillus, Bifidobacterium, Parasutterella, Helicobacter, Mucispirillum, Faecalibaculum, Muribaculum*, and *Acetatifactor*, displayed strong positive correlations with _L_-lactic acid, 3-hydroxybutyric acid, and allothreonine. Overall, *L. rhamnosus* microgels impacted the bacterial community and substantially altered the fecal metabolic profiling, affecting the host health.

### Intestinal Function and Morphology

**Figure 7:**
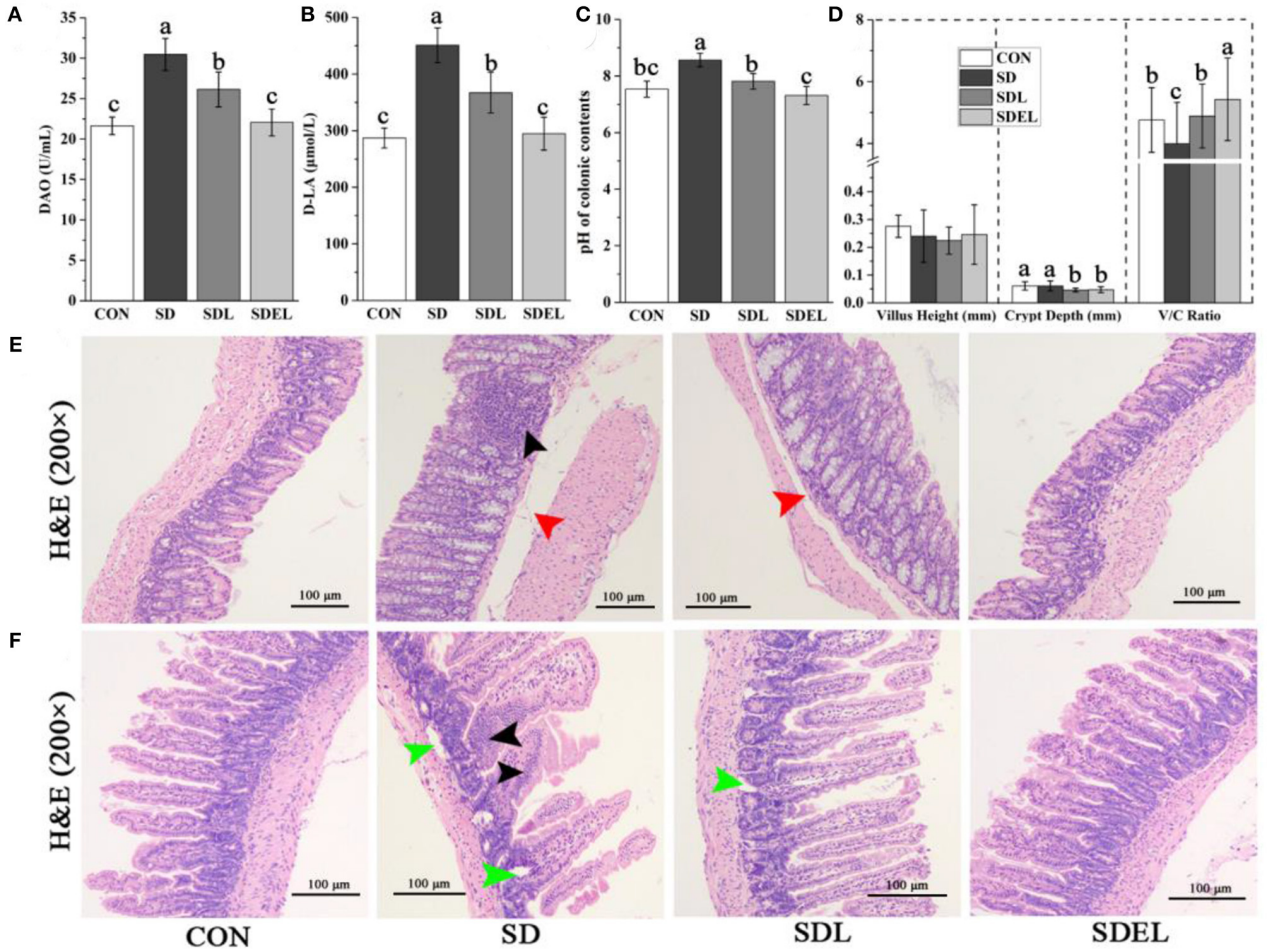
The colonic and small intestinal functions of the CON-, SD, SDL, and SDEL-fed mice. **(A)** Serum DAO activities and **(B)** D-LA concentrations indicate mice's colonic barrier function. **(C)** The pH of the colonic contents of the mice in different groups. **(D)** The villus height (V, mm), crypt depth (C, mm), and V/C ratio, which indicate the absorption function of the small intestine of the mice in different groups. **(E)** Colonic photomicrographs (100× magnification) of the mice in different groups. The black arrow indicates inflammatory cell infiltration; red arrows indicate edema in the submucosal layer. **(F)** Photomicrographs (100× magnification) of small intestinal villi of the mice in different groups. Black arrows and green arrows indicate infiltration of inflammatory cells and necrotized local muscularis. Data are expressed as mean ± SD; *n* = 8. Different letters were significantly different (*P* < 0.05).

The colonic barrier function and the small intestinal absorption functions were investigated in the CON-, SD-, SDL-, and SDEL-fed mice. Serum DAO activity and _D_-LA concentration were assessed to evaluate the effects of SDEL treatment on the intestinal permeability of SD-fed mice. Serum levels of DAO and _D_-LA were significantly higher in the SD group compared to the CON group (*P* < 0.05), indicating impaired colonic barrier function in the SD-fed mice (Figures 7A,B). In contrast, the levels of DAO and _D_-LA were significantly decreased in the SDL and SDEL groups compared to the SD group (*P* < 0.05), indicating reduced colonic barrier function following treatment of *L. rhamnosus* and its microgels. However, the DAO and _D_-LA levels in the SDEL group were significantly lower than the SDL group (*P* < 0.05), suggesting that *L. rhamnosus* microgels significantly reduced intestinal permeability in SDEL-fed mice. The pH of the colonic contents of the SD group (8.56 ± 0.24) was significantly different to that of the CON (7.54 ± 0.28), SDL (7.81 ± 0.28), and SDEL (7.31 ± 0.32) groups (*P* < 0.05). There was no statistically significant difference in pH value between the CON and SDEL groups (*P* > 0.05; Figure 7C). Furthermore, the villus height (V), crypt depth (C), and the V/C ratio in the small intestine were analyzed to evaluate the function of small intestinal absorption. The V/C ratio in the SDEL group was significantly higher than that in the CON, SD, and SDL groups (*P* < 0.05; Figure 7D), indicating that the *L. rhamnosus* microgels significantly restored the normal function of small intestinal absorption.

In both the CON- and SDEL-fed mice, colonic sections showed typical structures and boundaries of the mucosal, submucosal, muscularis, and serous layers. The mucosal glands were abundant, goblet cell structures were observed, and the bowel wall thickness was normal (Figure 7E). Conversely, in the colonic tissues of SD-fed mice, edema was found in the submucosal layer with an expanded gap (red arrow) and inflammatory cell infiltration (black arrow) was present between the mucosal glands (Figure 7E). The submucosa was slightly edematous (red arrow) in the colonic tissues of SDL-treated mice (Figure 7E). Furthermore, in both the CON and SDEL groups, the small intestine's submucosal, mucous, muscularis, and serous layers showed typical structures and boundaries (Figure 7F). However, infiltration of inflammatory cells (black arrows) and necrotized local muscularis (green arrows) into the small intestine was observed in the SD group. In the SDL-treated mice, the local muscularis had necrotized (green arrow; Figure 7F).

## Discussion

Excessive sodium intake has been identified as a risk factor for CKDs, the leading proponent of premature death worldwide (40). Recently studies have reported the association of salt-induced non-communicable diseases, such as CKDs (14), hepatic steatosis (25), and hypertension (26), with gut microbial dysbiosis. As the microbial habitat, the colon is the principal organ responsible for sodium homeostasis, and colonic epithelial cells help protect mammals against salt overload (41). The beneficial effects of probiotics have been confirmed in combating renal dysfunction by regulating the microbial community (24). Previous studies (42, 43) have confirmed that gavage with only *L. rhamnosus* can reversed the hepatic inflammation and slight renal injury associated with hyperuricaemia by regulating gut microbiota. However, the acidity of the gastrointestinal tract is a harsh environment that limits the mucoadhesion and colonization of alien probiotics. To date, microcapsules are still an ideal candidate to protect probiotics from the environmental stresses in food and medical industries (44). Zhuge et al. (45) delivered that *L. salivarius* in microgels could boost viability during transit through the gastrointestinal organs and attenuate liver injury by reducing inflammation and intestinal permeability. Zhao et al. (46) confirmed that probiotic (specifically, *Lactobacillus* and *Bacillus subtilis*) microcapsules could reduce inflammation, improve fat metabolism, and restore intestinal barrier functions, thus contributing to the treatment of metabolic syndrome *in vivo*. Nevertheless, the mechanism and effects of probiotics microgels on salt-induced hepatorenal failure have not been investigated. As *Lactobacillus* abundance is reduced in the gut by increased dietary sodium intake (26, 27), a correlation between *Lactobacillus* abundance and clinical outcomes of people consuming high salt diets is particularly interesting. The aims of the current study included encapsulating *L. rhamnosus* species in alginate and chitosan to prepare the microencapsulation for probiotic protection and evaluate the effects of *L. rhamnosus* microgels on salt-induced hepatorenal injury.

Both sodium alginate and chitosan are polyelectrolytes. Alginate extracted from brown seaweed (*Phaeophyceae*) is widely used for drug delivery due to its reliable water-holding capacity and heat stability. As linear copolymers composed of alternating sequences of β-D-mannuronic and α-L-guluronic acid residues (47), alginate will combine with multivalent cations (for example, Ca^2+^) to form gels or gentle matrices that can trap sensitive substances such as living probiotic bacteria (48, 49). Interestingly, an essential property of alginate is that they form insoluble gels formed at low pH conditions, but become soluble at neutral or higher pHs due to the ionically cross-linked carboxylate groups in the uronate blocks. This feature affords advantages in using of alginate as a support for probiotic delivery, avoiding the solubilization of gels in the gastrointestinal tract and providing adequate protection of bacteria within the acidic environment. Furthermore, the solubility of alginate at the gastrointestinal pH facilitates the release of viable cells into the gut (47). Alginate gel's mechanical and physicochemical properties can be further improved by forming an electrostatic complex membrane with a polycation, such as chitosan (50). Previous studies successfully encapsulated *L. plantarum, Escherichia coli*, and *B. longum* (28, 51, 52) into microgels containing alginate and chitosan. In the current study, similar strategies were used to prepare *L. rhamnosus* microgels using alginate and chitosan as the wall materials. The resulting effective gastric acid and bile acid resistances *in vitro* (Figure 2) shows uniformity in the reported encapsulation effects with previous studies (29, 53). The probiotic microgels had a defensive and reparative function in HSD-induced hepatorenal injury (Figures 3A–D).

Intestinal bacteria are either directly or indirectly involved in regulating the occurrence and development of hepatorenal injury through microbiome metabolite–host interactions. The structure of gut microbiota and its metabolites differed significantly between the CON and SD groups (Figures 4, 5). The α-diversity of bacterial communities were not confirmed, however, when comparing the CON- and SD-fed mice using the analyses of Shannon-Wiener and Simpson values (Figure 4D). Nonetheless, current study results showed that the SD group's microbiota was particularly enriched with the *Parvibacter, Campylobacter*, and *Marvinbryantia* genera. In contrast, other genera decreased, for example, *Lactobacillus, Oscillibacter, Roseburia*, and *Blautia*, compared with the CON group (Figures 4C,F), which is consistent with previous studies (25, 27). The abundance of *Lactobacillus* increased to varying degrees in the feces of SDL- and SDEL-treated mice and is particularly elevated raised to the highest abundance in the SDEL group (Figures 4C,F). Such outcomes confirm that alginate-chitosan encapsulation could exhibit superior protection of *L. rhamnosus* viability *in vivo* and ultimately colonization in the gastrointestinal tract.

Concomitantly, the well-known probiotic taxa, including *Bifidobacterium, Faecalibaculum*, and *Mucispirillum*, were observed in the SDEL group with more abundance (Figure 4C). These species had play a pivotal role in protection against CKD (54), colitis (55), and colorectal cancer (56), respectively. The enriched probiotics in the SDEL group were perhaps due to a lower pH value in the colonic environment of the SDEL-fed mice (Figure 7C), which stimulates probiotics growth, inhibits potential pathogenic growth, and modulates the immune system (57). As the metabolite of microbial fermentation, lactic acid is a chiral molecule that exists as _L_-lactic acid and _D_-lactic acid. _L_-lactic acid can be directly involved in human metabolism without any side effects. However, because mammalian tissues lack the enzyme metabolizing _D_-lactic acid, it has been reported that excessive levels of _D_-lactic acid may cause metabolic disorders and even acidosis (58). A previous study described *L. rhamnosus* as a homofermenter that solely produces _L_-lactic acid (59). _L_-lactic acid has displayed physiological and pathological functions *in vivo*, such as affecting of *Clostridium difficile* infections (60), a neuroprotective function (61), and regulating the lipid metabolism (62). In the present study, the abundance of _L_-lactic acid in the SDEL group, significantly increased by an average of 1.30 times more than in the SDL group (Figure 5C and Supplementary Table 1), which infers that *L. rhamnosus* may be more abundant than in the SDL group.

Additionally, microbial production of 3-hydroxybutyric acid has many potential applications as an intermediate for the synthesizing antibiotics, vitamins, aromatics, and pheromones in the host (63). 2-keto-L-gluconate (2-KLG), as a critical intermediate in vitamin C synthesis, could decrease the virulence of bacteria *in vivo* (64). Besides, the gut microbiota can convert primary bile acids—digestive chemicals produced by the liver—to secondary bile acids, including toxic DCA. DCA products pass into the liver through the hepatic portal vein and impair liver dysfunction (65). In the animal models, this metabolite of circulating bile acids was elevated in CKD and can induce vascular mineralization and osteogenic differentiation (66). In this study, the fecal metabolome results showed increased abundances of 3-hydroxybutyric acid and 2-KLG in the fecal samples of SDEL-treated mice. In contrast, the levels of cholic acid and DCA were significantly lowered compared with the feces of SD-treated mice (Figure 5C and Supplementary Table 1). Treatment of *L. rhamnosus* microgels led to the increased abundances of probiotic species including *Lactobacillus, Bifidobacterium, Mucispirillum*, and *Faecalibaculum*, disclosing strong positive correlations with _L_-lactic acid and 3-hydroxybutyric acid levels (Figure 6), which may exert beneficial effects on the hepatorenal health of the host. Furthermore, metabolites related to the central carbon metabolism in cancer, ABC transporters, arginine biosynthesis, tyrosine, alanine, aspartate and glutamate metabolism, and renal cell carcinoma pathways were significantly changed in the SDEL group relative to the SD group (Figure 5D). Accumulating evidence suggests that proteins associated with the central carbon metabolism in cancer (67), ABC transporters (68), and arginine biosynthesis (69) pathways are involved in the hepatorenal injury.

HSDs can decrease the *Lactobacillus* abundance in the murine gut, stimulate intestinal Th17 cells to respond, promote IL-17a production by intestinal epithelial cells, and induce inflammatory reactions (26, 70, 71). Meanwhile, IL-17a can govern the expression, pro-inflammatory effects, and tissue-protective properties of IL-22, and the presence or absence of IL-17a regulates the pathological vs. tissue-protective functions of IL-22 (72). This study confirmed that the intestinal *Lactobacillus* abundance significantly decreased and serum IL-17a and IL-22 levels significantly increased in SD-fed mice, while the *L. rhamnosus* microgels significantly increased intestinal *Lactobacillus* abundance and lowered the IL-17a and IL-22 levels in mice (Figures 3M,N, 4C). The intestinal functions, such as the colonic barrier function and the small intestine's absorption function, are highly associated with salt-induced non-communicable diseases (25, 26). Improving intestinal functions is a potential treatment for liver and kidney diseases (12, 73). With a focus on the microenvironment, it can be seen that *L. rhamnosus* microgels enhanced the colonic barrier function (Figures 7A,B) and the absorption function of the small intestine (Figure 7D), lowered the pH value of colonic contents (Figure 7C), and maintained the normal intestinal structures (Figures 7E,F) in the SD-fed mice. These actions indicated the positive effects of *L. rhamnosus* microgels in mitigating and repairing hepatorenal injury induced by high dietary salt levels.

## Conclusions and Perspectives

In conclusion, the encapsulation of *L. rhamnosus* into microgels loaded with alginate and chitosan shows excellent outcomes in enhancing probiotic viability in simulated gastrointestinal transit while also attenuating salt-induced hepatorenal injury through regulating the intestinal microbiota and metabolite profiles in mice. The data from the current study confirms the clinical potential of *L. rhamnosus* microgels in modulating and repairing hepatorenal and intestinal functions, gut microbiota and metabolism, and host immunity. Nevertheless, the mouse's average daily sodium intake was significantly higher than their tolerance, which likely does not occur in humans for extended periods (74). Human hepatorenal injury results from excessive salt consumption and other clinical and environmental factors over long periods (75). Thus, the effects of *L. rhamnosus* microgels on ameliorating clinical hepatorenal injury requires further clinical investigation to understand the entire scope of *L. rhamnosus* microgel treatments.

## Data Availability Statement

The datasets presented in this study can be found in online repositories. The names of the repository/repositories and accession number(s) can be found at: NCBI; PRJNA516991.

## Ethics Statement

The animal study was reviewed and approved by Shandong University of Traditional Chinese Medicine (No. SYXKLU20170022, Jinan, China). Written informed consent was obtained from the owners for the participation of their animals in this study.

## Author Contributions

ZZ and BC designed the research. ZZ, XC, ML, JL, WL, and ZC conducted the research. ZZ and XC wrote the manuscript and analyzed the data. BC and BY provided experimental equipment. All authors read and approved the final manuscript.

## Funding

This work received support from the National Natural Science Foundation of Shandong Province (Grant No. ZR2020QC237), National Undergraduate Innovation and Entrepreneurship Training Program in China (Grant No. S202010431032), National Key Research & Development Program in China (Grant No. 2019YFD1002704), and National Natural Science Foundation of China (Grant No. 81803864).

## Conflict of Interest

The authors declare that the research was conducted in the absence of any commercial or financial relationships that could be construed as a potential conflict of interest.

## Publisher's Note

All claims expressed in this article are solely those of the authors and do not necessarily represent those of their affiliated organizations, or those of the publisher, the editors and the reviewers. Any product that may be evaluated in this article, or claim that may be made by its manufacturer, is not guaranteed or endorsed by the publisher.

## Abbreviations

CKD: chronic kidney disease
CFU: colony-forming units
CON: control
DCA: deoxycholic acid
HSD: high-salt diet
SDL: HSD with *Lactobacillus rhamnosus* cells
SDEL: HSD plus *L. rhamnosus* microgels
SIF: simulated intestinal fluid
SGF: simulated gastric fluid
_D_-LA_D_-lactate, DAO: diamine oxidase
IL-17a: interleukin-17a
IL-22: interleukin-22
OUT: operational taxonomic units
ELISA: enzyme-linked immunosorbent assay
BUN: blood urea nitrogen
CRE: creatinine
UA: uric acid
GLC: glucose
ALT: glutamic-pyruvic transaminase
AST: glutamic-oxalacetic transaminase
ALP: alkaline phosphatase
TG: triglycerides
LEfSe: linear discriminant analysis effect size
PCoA: principal co-ordinates analysis.
