# Supplementary figures and images for: Lactobacillus rhamnosus Encapsulated in Alginate/Chitosan Microgels Manipulates the Gut Microbiome to Ameliorate Salt-Induced Hepatorenal Injury
Source: Front Nutr. 2022 Apr 14;9:872808. doi: 10.3389/fnut.2022.872808 (PMC9047548; doi:10.3389/fnut.2022.872808)

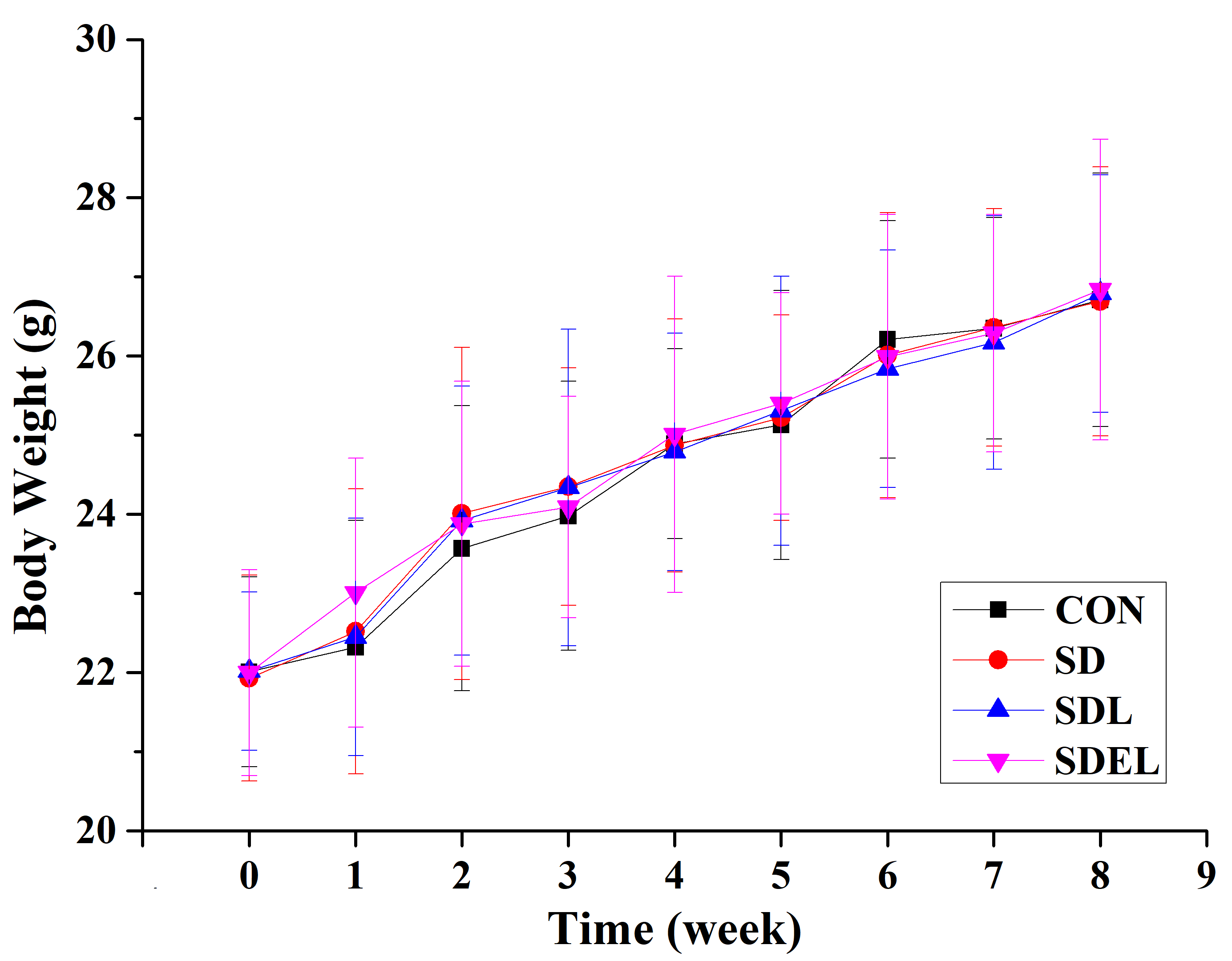

Supplement: Supplementary file 2 [file Image_1.TIF]
